# Role of Fibrinolytic Enzymes in Anti-Thrombosis Therapy

**DOI:** 10.3389/fmolb.2021.680397

**Published:** 2021-05-28

**Authors:** Farwa Altaf, Shourong Wu, Vivi Kasim

**Affiliations:** ^1^ The Key Laboratory of Biorheological Science and Technology, Ministry of Education, College of Bioengineering, Chongqing University, Chongqing, China; ^2^ The 111 Project Laboratory of Biomechanics and Tissue Repair, College of Bioengineering, Chongqing University, Chongqing, China

**Keywords:** thrombosis therapy, thrombolytic drugs, plasminogen activators, proteases, fibrinolytic enzymes

## Abstract

Thrombosis, a major cause of deaths in this modern era responsible for 31% of all global deaths reported by WHO in 2017, is due to the aggregation of fibrin in blood vessels which leads to myocardial infarction or other cardiovascular diseases (CVDs). Classical agents such as anti-platelet, anti-coagulant drugs or other enzymes used for thrombosis treatment at present could leads to unwanted side effects including bleeding complication, hemorrhage and allergy. Furthermore, their high cost is a burden for patients, especially for those from low and middle-income countries. Hence, there is an urgent need to develop novel and low-cost drugs for thrombosis treatment. Fibrinolytic enzymes, including plasmin like proteins such as proteases, nattokinase, and lumbrokinase, as well as plasminogen activators such as urokinase plasminogen activator, and tissue-type plasminogen activator, could eliminate thrombi with high efficacy rate and do not have significant drawbacks by directly degrading the fibrin. Furthermore, they could be produced with high-yield and in a cost-effective manner from microorganisms as well as other sources. Hence, they have been considered as potential compounds for thrombosis therapy. Herein, we will discuss about natural mechanism of fibrinolysis and thrombus formation, the production of fibrinolytic enzymes from different sources and their application as drugs for thrombosis therapy.

## Introduction

In 2017, cardiovascular diseases (CVDs) caused 17.7 million deaths globally (CVDs) (Yusuf et al., 2020). Thrombosis is the formation of blood clot in both artery and venous arterial or venous circulation. Arterial thrombosis is caused mainly by accumulation of platelets, fibrin, and thrombin, which could leads to the formation and spreading of atherosclerotic plaque (Boos and Lip, 2006), and is the major cause of most cases of heart attack (myocardial infarction) (Mackman, 2008).

Fibrinogen is a large soluble plasma glycoprotein produced and released by the liver, and converted into polymeric fibrin by thrombin during damage to vascular system. Initiation of the blood clotting process occurs at the wound site when platelets are aggregated and a proteolytic cascade mechanism to convert fibrinogen into fibrin is started (Doolittle, 2010; Weisel and Litvinov, 2017). Normally these clots are hydrolyzed by plasmin; however, aberrant control of the hydrolysis process could cause hyperfibrinogenemia, which, subsequently, leads to a variety of thrombotic diseases such as stroke (Danesh et al., 2005; Chelluboina and Vemuganti, 2019), abdominal aortic aneurysm (Parry et al., 2009; Kapetanios et al., 2019), peripheral vascular disease (Altes et al., 2018), pulmonary embolism (Klovaite et al., 2013), and cardiovascular disease (CVD) (Tatli et al., 2009; Liu et al., 2020).

Various anti-coagulants like heparin, fondaparinux, idraparinux (Gross and Weitz, 2008), warfarin (Kneeland and Fang, 2010), and rivaroxaban have been used for treating thrombosis; however, they could also cause side effects such as bleeding, which is the major problem in all anti-coagulants, hemorrhages (Stam et al., 2002), and long term adherence. Furthermore, solving uncertainty about their dosing and expensive process are also in urgent (Bauer, 2013; Burgazli et al., 2013). Thrombolytic agents such as urokinase and plasminogen activators have been extensively used for treating thrombosis; however, in addition to their high costs, they might also caused internal hemorrhage within the intestinal tract when administered orally (Flemmig and Melzig, 2012; Kotb, 2012). Likewise, anti-platelets such as aspirin, prasugrel, and ticagrelo which are used to prevent clot formation, could also bleedings, including intracranial haemorrhage, skin bruising, and gastrointestinal bleeding (Swan et al., 2020). These draw backs have evoked researchers to look for safer and cheaper resources (Flemmig and Melzig, 2012; Kotb, 2012; Kotb et al., 2015).

Fibrinolytic enzymes are involved in the degradation of fibrin clots, by either catalyzing fibrin degradation process or by transforming the inactive plasminogen into active plasmin, thus re-establishing the normal blood vascular architecture (Krishnamurthy et al., 2018). They are largely proteases, which are involved in the total hydrolysis of proteins, and can be produced from all living cells especially bacteria. On the basis of their site of actions, proteases could be divided into two groups: endopeptidases and exopeptidases; while on the bases of the distinct functional groups located at the active site, they could be classified into five types, namely cystein proteases, serine proteases, threonine proteases, metalloproteases and aspartic proteases (Flemmig and Melzig, 2012). Fibrinolytic enzymes have been discovered from different sources such as microorganisms, plants, animals and fermented products, and among them, the most important sources are micoorganisms, especially genous *Bacillus* from the traditional fermented foods (Mine et al., 2005). Due to their property of dissolving the thrombus by directly degrading the fibrin at high rate, microbial fibrinolytic enzymes have the potential to be used as drugs for treating thrombosis and other related diseases (Kotb, 2012). In this review, we will outline the mechanisms of fibrinolysis, thrombus formation, isolation of fibrinolytic enzymes from different sources and their role in thrombus degradation.

## Thrombus Formation

Injury to the wall of blood vessel and eruption of blood from the circulation triggers the onset of thrombus formation through the processes including formation of atherosclerotic plaque, activation of platelets and coagulation pathways (Furie and Furie, 2008; Asada et al., 2018). The composition of thrombi is different in arteries and veins, venous thrombi are red clots due to abundance of fibrin and red blood cells, while arterial thrombi are white clots as they mainly composed of platelets aggregates (Franchini and Mannucci, 2008).

Activation of endothelial cells upon aggregation of cholesterol containing low density lipoprotein (LDL) serves as the initiation atherosclerotic process. Endothelial cells express chemokines and leukocyte adhesion molecules that boost the recruitment of T cells and monocytes to the site. Monocytes will then differentiate into macrophages and this will upregulate the expression level of pattern recognition receptors, including scavenger and toll-like receptors (TLRs) (Lippi et al., 2011). These pattern recognition receptors will in turn enhance the uptake of lipoproteins, which subsequently lead to the generation of foam cells. Binding of ligand to TLRs transfer macrophage-activating signals that results in the discharge of cytokines, proteases and other vasoactive molecules (Hodgkinson and Ye, 2011). T lymphocytes produce proinflammatory cytokines that interact with local antigens and support T-helper-1 cell responses within the atherosclerotic lesion, which further promote the plaque growth and enhance local inflammation (Lundberg and Hansson, 2010). Prolonged local inflammation results in the disruption and proteolysis of atherosclerotic plaque, leading to plaque erosion and rupture (Lippi et al., 2011).

Atherosclerotic plaques contain foam cells, tissue factor (TF), lipid droplets, necrotic cell debris and matrix constituents as well as other platelets activating molecules such as fibrinogen, fibrin, vitronectin, thrombospondin, von Willebrand factor (VWF, a plasma protein carrier for factor VIII essential for adhesion of platelets to the vessel wall), fibronectin, stromal derived factor 1, various types of collagens, LDLcholesterol sulfate and oxidized LDL. These molecules actively stimulate adhesion and accumulation of platelets along with emission of their heavy granules. Human atherosclerotic plaques induce the formation of arterial thrombi through a complex process involving platelets aggregation and accumulation, as well as the activation of blood coagulation, which subsequently leads to the formation of thrombus (Reininger Armin et al., 2010; Lippi et al., 2011).

Platelet aggregate formation is initiated by the interaction of platelet surface receptors glycoprotein Ib-IX-V (GPIb-IX-V, cluster of adhesive receptors on platelets) and glycoprotein VI (GPVI, a collagen receptor) with collagenous plaque components such as VWF and collagen, respectively. Then a shift in the conformation of integrins on the platelets triggers the initial phase of platelet accumulation, involving various factors such as glycoprotein IIb/IIIa (GPIIb/IIIa, also known as GP_α_I/βIII or integrin α_3_β_2_), glycoprotein-receptor-Ia/IIa (GPIa/IIa also known as integrin α_2_β_1_ for collagen binding), adenosine diphosphate (ADP), and thromboxance A_2_ (TXA_2_). GIIb/IIIa is a fibrinogen receptor attaches with VWF and fibrinogen and mediates tight adhesion, spreading, coagulation activity, and accumulation of platelets; while GPIa/IIa mediates collagen–platelet binding under low-shear-rate conditions, such as those near an atherosclerotic plaque, and strengthen the connection between collagen and glycoprotein VI (GPVI). Then ADP promotes platelets to undergo shape change, leading to the release of granule contents and platelets aggregation. Furthermore, thromboxance A2 (TXA_2_) acts as a positive feedback regulator that activates and recruits more platelets to the main hemostatic plug (Furie and Furie, 2008; Reininger Armin et al., 2010; Lippi et al., 2011).

After activation and recruitment of platelets, blood coagulation will be initiated by binding of tissue factor to factor VIIa (FVIIa) and thrombin burst, which encompasses the transformation of inactive proenzymes to into their respective serine proteases in a sequential manner, leading to thrombin generation (Reininger Armin et al., 2010). Thrombin burst will then be formed in the presence of factor XI (FXI), factor X (FX), and factor IX (FIX), which act as a physiological amplificatory mechanism propagating thrombin generation. During this, fibrin produced by the enzymatic action of factor X on fibrinogen and TAFI (thrombin-activatable fibrinolysis inhibitor) will also downregulate fibrinolysis by weakening the plasmin mediated fibrin breakdown to promote thrombin formation (Lippi et al., 2011). Together, thrombus formation is a complex, multifactors-regulated process involving the synthesis of atherosclerotic plaques, platelets aggregation, and blood coagulation.

## Fibrinolysis

Fibrinolysis is the enzymatic breakdown of fibrin network of blood clots by fibrinolytic enzymes (Bannish et al., 2017). This process is regulated by two steps: first, tissue plasminogen activator (tPA) and urokinase plasminogen activator (uPA) convert plasminogen into serine protease plasmin and second, fibrin is broken down into fibrin degradation products, thus restoring the bood flow by dissolving the thrombus (Gue and Gorog, 2017). These processes are regulated by the engagement of substrates, cofactors, activators, receptors and inhibitors, which work synergistically to guarantee the fluidity of blood (Cesarman-Maus and Hajjar, 2005; Bannish et al., 2017).

The major fibrinolytic protease is plasmin formed by conversion of plasminogen by both tissue plasminogen activator (tPA) and urokinase plasminogen activator (uPA). Through a positive feedback mechanism, plasmin cleaves the single chain of both uPA and tPA into two more active polypeptide chains. Plasmin generation is increased by the degradation of fibrin, the major substrate of plasmin, by binding both tissue plasminogen activator and plasminogen on its surface (Cesarman-Maus and Hajjar, 2005). In the absence of fibrin, tPA acts as a weak activator of plasminogen, while the presence of fibrin significantly increases the affinity between tPA and plasminogen, and thus enhances the catalytic efficiency of tPA in activating plasminogen. After being formed, plasmin produces soluble degradation products by cleaving the fibrin. The “kringles” 1 and 4 domains of plasminogen and 2 of tPA carry lysine binding sites, which enhance its binding to fibrin, thus causing the increased plasmin production and removal of fibrin (Kolev and Machovich, 2003; Cesarman-Maus and Hajjar, 2005).

In healthy person, the regulation of fibrinolysis is achieved by certain regulators including fibrinolysis inhibitors and plasminogen activator inhibitor. Fibrinolysis inhibitors, such as thrombin activatable fibrinolysis inhibitor (TAFI), and lysin analogues including tranexamic acid and epsilon aminocaproic acid (Bridge et al., 2014). TAFI reduces the fibrinogen and fibrin binding, α2-antiplasm forms complex with plasmin, increases adsorption of PLG on fibrin and crosslinks FXIIIa, a clotting factor that produce the cross-links between fibrin strands and inhibitors such as TAFI and α2-antiplasm; while PAI-1, a plasminogen activator inhibitor whose production and binding ability to plasminogen could be enhanced by lipoprotein (a), inhibits uPA and tPA (Gue and Gorog, 2017).

Together, fibrinolysis is crucial for anti-thrombin therapy, and plasmin is the major enzyme responsible for fibrinolysis. While many fibrinolytic enzymes have been used for clinical treatment, natural sources for obtaining this enzyme as described below might be potential as sources for fibrinolytic enzyme due to their activity and low production costs.

## Microorganisms as Sources of Fibrinolytic Enzymes

Microorganisms are the most important and cheap source of fibrinolytic enzymes, and many of them, such as Streptokinase and Staphylokinase, which were isolated from *Streoptococcus hemolyticus* and *Streptococcus aureus,* are effective in thrombolytic therapy (Collen and Lijnen, 1994). Since then, many fibrinolytic enzymes from various microbial and non-microbial sources have been discovered in succession.

Bacteria that belong to genus *bacillus* are the most important source of fibrinolytic enzymes (Table 1). Since their discovery, many studies have been performed to optimize the production conditions of fibrinolytic enzyme to increase their yield by using different methods of fermentation, including solid-state, and submerged fermentation (Vijayaraghavan et al., 2017; Al Farraj et al., 2020; Che et al., 2020), mutagenesis (Naveena et al., 2012; Raju and Divakar, 2013a), recombination (Che et al., 2020), statistical approaches (Chandramohan et al., 2019; Joji et al., 2019) and gene cloning techniques (Nguimbi et al., 2014). Al Farraj *et al.* enhanced the production of fibrinolytic enzyme from a new bacterium *Bacillus flexus* obtained from marine environments by using statistical approach, two-level full factorial design 2^5^ and response surface methodology, to optimize the conditions for the production of fibrinolytic enzyme using solid state fermentation process. Under this optimized condition, they improved the fibrinolytic enzyme output up to 3.5 fold (Al Farraj et al., 2020). Submerged fermentation has also been chosen for the discovery of fibrinolytic enzyme and for improving the production efficacy. Anusree *et al.* optimized the production of fibrinolytic enzyme isolated from *Serratia rubidaea* KUAS001 by using submerged fermentation (Anusree et al., 2020). Staphylokinase (SAK) from *Staphylococcus aureus* GH38 was also screened by using submerged fermentation (Noori and Aziz, 2020); while Pan *et al.* reported for the first time the use of non-sterile submerged-fermentation to reduce the production cost of fibrinolytic enzyme from *Bacillus subtilis* D21-8 (Pan et al., 2019a). Furthermore, use of different statistical tools such as Box-Benhken design (Kumar et al., 2018), two-level full factorial design (2^5^) (Vijayaraghavan et al., 2016a; Vijayaraghavan et al., 2016b), response surface methodology (RSM), central composite design (CCD) and artificial neural network (ANN) (Chandramohan, et al., 2019; Joji et al., 2019), as well as orthogonal experiment (Wu et al., 2019) have been reported to optimize the concentration of media components used for fermentation to obtain fibrinolytic enzymes in order to enhance their production yield.

**TABLE 1:** Fibrinolytic enzymes produced by bacteria from different sources.

Bacteria	Source	Name of enzyme	eferences
*Bacillus flexus*	South West coast of India	N.A.	Al Farraj et al. (2020)
*Pseudomonas aeruginosa* KU1	Marine sediments of Ezhara beach	*Pseudomonas aeruginosa* KU1 (PEKU1)	Kumar et al. (2020)
*Serratia rubidaea*	Marine samples (sediments, water)	*Serratia rubidaea* KUAS001	Anusree et al. (2020)
*Staphylococcus aureus* GH38	From a patient suffering from burns	Staphylokinase (SAK)	Noori and Aziz (2020)
*Fictibacillus* sp. strain SKA27	Sand, seashells, and marine water	N.A.	Joji et al. (2019)
*Bacillus subtilis* WR350	Through UV mutagenesis of *B. subtilis* HQS-3	N.A.	Wu et al. (2019)
*Bacillus subtilis* D21-8	Through UV mutagenesis of *B. subtilis* HQS-3	N.A.	Pan et al. (2019a), Pan et al. (2019b)
*Bacillus cereus* RSA1	From soil	Protease	Sharma et al. (2020)
*Alcaligenes aquatilis* PJS_1	Soil from slaughter houses	N.A.	Prabhu et al. (2020)
*Bacillus pseudomycoides* strain MA02	From poultry slaughter house soils	Protease	Chandramohan et al. (2019)
*Pseudomonas aeruginosa* KU1	From marine sediments	N.A.	Kumar et al. (2018)
*Stenotrophomonas* sp. KG-16-3	Soil samples from different habitats (dairy, garbage dump, slaughter house)	Protease	Taneja et al. (2019)
*Stenotrophomonas maltophilia* Gd2	From soil	Protease	Khursade et al. (2019)
*Bacillus tequilensis*	From soil	N.A.	Xin et al. (2018)
*Bacillus subtilis*	From Egyptian soil	N.A.	Moharam et al. (2019)
*Serratia marcescens* subsp. *sakuensis*	Sea water	N.A.	Krishnamurthy et al. (2017)
*Serratia* sp KG-2-1	Garbage dump soil sample	Protease	Taneja et al. (2017)
*Bacillus cereus* SRM-001	Blood-laden soil of a chicken waste-dump yard	N.A.	Narasimhan et al. (2018)
*Bacillus subtilis*	N.A.	Bacillopeptidase CFR5 (BPC)	Sharmila and Venkateswaran (2017)
Actinomycete	From Microbial Biotechnology Research Laboratory (MBRL) culture collection	N.A.	Ningthoujam and Thokchom (2016)
*Streptomyces* sp	Amazonian lichens	Serine protease	Silva et al. (2016)
*Bacillus Amyloliquefaciens* UFPEDA 485	Cultures Collections of Department of Antibiotics	Serine-metalloprotease	de Souza et al. (2016)
N.A.	Sea water	Metalloprotease	Velumani, (2016)
*Bacillus* sp. IND12	Soil, fish, and rice	N.A.	Vijayaraghavan et al. (2017)
*Shewanella* sp. IND20	From fish *Sardinella longiceps*	N.A.	Vijayaraghavan and Prakash Vincent (2015)
*Bacillus cereus* SRM-001	Blood-laden soil of a chicken dump yard	N.A.	Narasimhan et al. (2015)
*Proteus penneri* SP-20	Soil from slaughter house waste	Protease	Jhample et al. (2015)
*Bacillus licheniformis*	Soil samples from hot Spring	Protease	Shukor et al. (2015)
*Streptomyces* sp. P3	From air-dried soils	FSP3: serine protease	Cheng et al. (2015)
*Brevibacillus brevis* strain FF02B	N.A.	Brevithrombolase: serine protease	Majumdar et al. (2015)
*Streptomyces* sp. DPUA 1576	Amazon lichens	N.A.	Silva et al. (2015)
*Pseudoalteromonas* sp. IND11	Fish scales	N.A.	Vijayaraghavan and Vincent (2014)
*Bacillus subtilis* I-2	Soil from slaughter-houses, dairy, domestic garbage and compost	Protease	Bajaj et al. (2014)
*Bacillus* sp. UFPEDA 485	Collection of Microorganisms	Metalloprotease	Sales et al. (2013)
*Bacillus subtilis* GXA-28	China Center for Type Culture Collection	N.A.	Zeng et al. (2013)
*Bacillus subtilis* HQS-3	Marine sample	Serine metalloprotease	Huang et al. (2013)
*Bacillus cereus* NS-2	From slaughter houses, dairy and domestic garbage)	Protease	Bajaj et al. (2013)
*Bacillus cereus* GD 55	From soils	Protease	Raju and Divakar (2013a)
*B. cereus, B. circulans, P. aeruginosa, P. fluorescens, E.coli*	From slaughter houses of beef, chicken and fish	Protease	Raju and Divakar (2013b)
*Streptomyces* sp. XZNUM 00004	From rhizosphere soil of *P. sibiricum*	*Streptomyces* fibrinolytic enzyme-1 (SFE1)	Ju et al. (2012)
*Streptomyces venezuelae*	From marine water	Thrombinase	Naveena et al. (2012)
*B. subtilis*, *streptococci*, *Pseudomonas* sp	From soil sample, blood and biomass from infected throat, human urine respectively	Nattokinase, Streptokinase and Urokinase	Dubey et al. (2011)
*Bacillus subtilis* TKU007	From soil	*Bacillus subtilis* nattokinase-1 BSN1	Wang et al. (2011)
*Streptomyces* sp. CS624	From soil	FES624: chymotrypsin-like serine metalloprotease	Mander et al. (2011)
*Paenibacillus polymyxa* EJS-3	From tissues of *Stemona japonica*	*Paenibacillus polymyxa* EJS-3 fibrinolytic enzyme-1 (PPFE-I)	Lu et al. (2010)
*Bacillus* sp. *strain* AS-S20-I	N.A.	Bafibrinase: Serine protease	Mukherjee et al. (2012)

N.A., not available.

Besides optimizing the fermentation condition, gene cloning, mutagenesis and DNA recombination technology have also been used to enhance fibrinolytic enzyme production. Using gene cloning, Yao et al. obtained a recombinant fibrinolytic enzyme with significantly higher fibrinolytic activity, rAprEBS15, isolated from *Bacillus pumilus* BS15 (Yao et al., 2018). Using codon optimization, gene dosage and process optimization, Che *et al.* expressed and secreted fibase, a fibrinolytic enzyme from marine *Bacillus subtilis*, and significantly enhanced its activity (Che et al., 2020).

Other microorganisms such as algae, fungi, and fermented foods have also been used to obtain fibrinolytic enzymes by either using different fermentation methods or aqueous two phase systems (Nascimento et al., 2016; Meshram et al., 2017). Algae are sources for fibrinolytic enzymes that belong to proteases, metalloproteases, and tissue-type plasminogen activator (Choi et al., 2013; Kim et al., 2013; Moon et al., 2014; Kang et al., 2016; Min et al., 2016; e et al., 2017; Silva et al., 2018; de Barros et al., 2020) (Table 2); while fungi are important sources for fibrinolytic enzymes that belong to proteases, metalloproteases and metallo-endopeptidase (Rovati et al., 2009; Choi et al., 2011; Kim et al., 2011; Liu et al., 2012; Palanivel et al., 2013; Ahmad et al., 2014; Liu et al., 2014; Kotb et al., 2015; Shukor et al., 2015; Meshram et al., 2016; Nascimento et al., 2016; Meshram et al., 2017; Liu et al., 2017; da Silva et al., 2019) (Table 3).

**TABLE 2:** Fibrinolytic enzymes produced by algae from different sources.

Algae	Source	Name of enzyme	References
*Arthrospira platensis*	Culture collection of Algae	Serine metalloprotease	de Barros et al. (2020)
*Chlorella vulgaris* UTEX 1803	From University of Texas, Austin	Protease	Silva et al. (2018)
*Chlorella Vulgaris*	University of Texas, Austin	Protease	Páblo et al. (2017)
*Undaria pinnatifida*	Sigma (St. Louis, MO, United States)	Tissue-type plasminogen activator	Min et al. (2016)
*Ulva pertusa*	Coastal area	Ulvease: serine protease	Kang et al. (2016)
*Lyophyllum shimeji*	Yedang Mushroom Co	α chymotrypsin like serine metalloprotease	Moon et al. (2014)
*Costaria costata*	Coastal area	Serine protease	Kim et al. (2013)
*Codium fragile*	Coastal area	Codiase: serine protease	Choi et al. (2013)

**TABLE 3:** Fibrinolytic enzymes obtained from fungi from different sources.

Fungi	Source	Name of enzyme	References
*Mucor subtilissimus* UCP 1262	N.A.	Protease	da Silva et al. (2019)
*Cordyceps militaris*	N.A.	Serine protease	Liu et al. (2017)
*Xylaria curta*	Stem of plant: *Catharanthus roseus*	Protease	Kim et al. (2011); Meshram et al. (2017)
*Mucor subtillissimus* UCP 1262	Soil	Protease	Nascimento et al. (2015)
*Aspergillus japonicum* KSS 05	Soil	N.A.	Shukor et al. (2015)
*Pleurotus ostreatus*	Microbiological Culture collection Center	Metallo-endopeptidases	Liu et al. (2014)
*Coprinus comatus* YY-20	N.A.	N.A.	Liu et al. (2012)
*Cordyceps militaris*	N.A.	Chymotrypsin-like serine metalloprotease	Choi et al. (2011)
*Mucor subtilissimus UCP 1262*	Soil	Protease	Nascimento et al. (2016)
*Xylaria curta*	*Cathranthus roseus twigs*	Xylarinase: metalloprotease	Meshram et al. (2016)
Endophytic Fungi	*Hibiscus* leaves	Protease	Ahmad et al. (2014)
*Trichoderma, Aspergillus, Penicillium, Rhizopus* and *Mucor*	Alkaline soil	Protease	Palanivel et al. (2013)
*Aspergillus brasiliensis* AUMC 9735	Dairy products, meats, soybean powders, soil and water samples	Metalloprotease	Kotb et al. (2015)
*Paecilomyces tenuipes*	Culture collection of DNA Bank of Mushrooms	*Paecilomyces tenuipes* entomopathogenic fibrinolytic protease: PTEFP	Kim et al. (2011)
*Bionectria* sp.	Las Yungas rainforest	Serine-proteases	Rovati et al. (2009)

N.A., not available.

Fermented foods are also known as sources for fibrinolytic enzymes (Table 4). Fermented soybeans have been consumed as traditional foods in many Asian countries, and indeed, they are sources of nattokinase and other fibrinolytic enzymes. These includes natto, a Japanese traditional fermented soybean obtained from fermentation with *Bacillus subtilis* G8 and *Bacillus subtilis* (Chang et al., 2012; Lucy et al., 2019), moromi, oncom and gambus (Indonesian traditional fermented soybean obtained from fermentation with *Bacillus cereus*, *Bacillus subtilus*, *Bacillus pumilus*, and *Stenotrophomonas* sp, respectively) (Afifah et al., 2014; Nailufar et al., 2016; Stephani et al., 2017; Syahbanu et al., 2020) as well as douchi and doufuru (Chinese traditional fermented soybean obtained from fermentation with *Bacillus subtilis* DC27, *Bacillus subtilis* LD-8547, *Bacillus subtilis* DC33, and *Bacillus subtilis FR-33*) (Ok and Choi, 2005; Wang et al., 2006; Jo et al., 2011; Wei et al., 2011; Yuan et al., 2012; Chen et al., 2013; Heo et al., 2013; Park et al., 2013; Gad et al., 2014; Thokchom and Joshi, 2014; Bi et al., 2015; Huy et al., 2016; Liu et al., 2016; Nailufar et al., 2016; Hu et al., 2019; Ito, 2020; Syahbanu et al., 2020). Moreover, fibrinolytic enzymes could also be obtained from other fermented products, such as rice fermented with *Xanthomonas oryzae* IND3, *Bacillus cereus* IND5, *Bacillus halodurans* IND18, *Bacillus* sp. IND7 and *Aspergillus oryzae* KSK-3 (Shirasaka et al., 2012; Biji et al., 2016; Vijayaraghavan et al., 2016a; Vijayaraghavan et al., 2016b; Vijayaraghavan et al., 2019), seafood fermented with *Bacillus* sp, *Bacillus. velezensis* BS2, *Bacillus pumilus* BS15 and *Bacillus coagulans* (Anh et al., 2015; Prihanto and Firdaus, 2019; Yao et al., 2019; Kim et al., 2020) and liquors obtained from fermentation with *Bacillus amyloliquefaciens*, *Bacillus licheniformis* and Bacillus-Genus (Lapsongphon et al., 2013; Johnson et al., 2015; SolokaMabika et al., 2020).

**TABLE 4:** Fibrinolytic enzymes from microorganisms food products.

Microorganisms	Name of enzyme	Food	References
*Bacillus cereus*, *B. subtilis*, *B. cereus*.	Protease	Indonesian fermented soybean: moromi	Syahbanu et al. (2020)
Bacillus-Genus	N.A.	Brazzaville: Squash	SolokaMabika et al. (2020)
N.A.	N.A.	Japanese fermented food: funazushi	Ito (2020)
N.A.	JP-I (Jotgal protease-I) and JP-II	Korean traditional fermented food: Jotgal	Kim et al. (2020)
*Bacillus subtilis* DC27	Douchi fibrinolytic enzyme: DFE27	Chinese fermented soybean food: Douchi	Hu et al. (2019)
*Bacillus subtilis* G8	N.A.	Japanese Fermented Natto Soybeans	Lucy et al. (2019)
*Bacillus. velezensis* BS2	N.A.	Korean fermented seafood: sea squirt (munggae) jeotgal	Yao et al. (2019)
*Xanthomonas oryzae* IND3	Protease	Fermented rice	Vijayaraghavan et al. (2019)
*Bacillus pumilus* BS15	N.A.	Korean fermented sea food: jeotgal	Yao et al. (2018)
*Stenotrophomonas* sp	Extracellular protease	Indonesian fermented soybean: Oncom	Stephani et al. (2017)
*Stenotrophomonas* sp	N.A.	Indonesian Fermented Soybean: Oncom	Nailufar et al. (2016)
*Bacillus amyloliquefaciens*	N.A.	Vietnamese soybean-fermented products	Huy et al. (2016)
Fungus (*Neurospora sitophila*)	N.A.	Food-grade fungus	Liu et al. (2016)
*Bacillus* sp. IND7	Protease	Cooked rice	Vijayaraghavan et al. (2016a)
*Bacillus cereus* IND5	N.A.	Fermented rice	Biji et al. (2016)
*Bacillus halodurans* IND18	N.A.	Fermented rice	Vijayaraghavan et al. (2016b)
*Bacillus subtilis* XZI125	N.A.	Fermented soybean meal	Bi et al. (2015)
N.A.	N.A.	Indonesian palm wine: tuak	Johnson et al. (2015)
*Bacillus* sp	N.A.	Fermented Shrimp Paste	Anh et al. (2015)
*Lactococcus lactis*, *Vagococcus lutrae*, *V. fluvialis*, *Weissella thailandensis*, *B methylotrophicus*	N.A.	Fermented soybean foods of North-East India	Thokchom and Joshi (2014)
*Bacillus pumilus*	N.A.	Indonesian fermented soybean cake: gembus	Afifah et al. (2014)
*Bacillus amyloliquefaciens, Bacillus licheniformis*	Nattokinase	Spoilt milk and soy flour	Gad et al. (2014)
*Bacillus coagulans*	N.A.	Indonesian fermented fish products: Terasi and Jambal roti	Prihanto and Firdaus, (2019)
*Bacillus amyloliquefaciens CB1*	AprECB1	Korean fermented soy food: cheonggukjang	Heo et al. (2013)
*Bacillus subtilis FR-33*	N.A.	Chinese soy cheese doufuru	Chen et al. (2013)
*Virgibacillus* sp. SK37	Proteinase	Brewery Yeast Sludge	Lapsongphon et al. (2013)
*Bacillus subtilis WRL101*	Nattokinase WRL101	Doenjang	Park et al. (2013)
*Bacillus subtilis*	Subtilisin-like serine protease	Fermented natto-red bean	Chang et al. (2012)
*Bacillus subtilis* LD-8547	Douchi fibrinolytic enzyme (DFE)	Chinese soybean-fermented food: Douchi	Yuan et al. (2012)
*Aspergillus oryzae* KSK-3	Serine protease	Rice koji	Shirasaka et al. (2012)
*Bacillus amyloliquefaciens* MJ5-41	AprE5-41	Korean fermented soy product: Meju	Jo et al. (2011)
*Bacillus amyloliquefaciens*	N.A.	Chinese soybean paste	Wei et al. (2011)
*Bacillus subtilis* DC33	Subtilisin-like serine protease: subtilisin FS33	Chinese soybean-fermented food: Ba-bao Douchi	Wang et al. (2006)
*Bacillus* sp	N.A.	Korean fermented soybean paste	Ok and Choi (2005)

N.A., not available.

## Macro-Organisms as Sources of Fibrinolytic Enzymes

While microorganisms are the main sources for fibrinolytic enzymes, they have also been isolated from non-microbial organisms such as plants, parasites, snake venoms and earthworms. Plants are also important sources of fibrinolytic enzymes, especially proteases (Table 5). Cysteine protease could be extracted from the leguminous *Gliricidia sepium* PBSGS using aqueous two-phase systems of sodium phosphate and PEG (da Silva et al., 2020); while serine proteases, which have the potential to degrade α, β and γ chains of fibrinogen and fibrin clots, could be extracted from different plants, including latex of *Ficus carica*, leaves of *Cnidoscolus urens* (L.), leaves of *Petasites japonicas*, latex of *Artocarpus heterophyllus*, leaves of *Aster yomena*, leaves of *Allium tuberosum* and latex of *Euphorbia hirta* (Chung et al., 2010a; Chung et al., 2010b; Patel et al., 2012; Siritapetawee et al., 2012; Choi et al., 2014; De Menezes et al., 2014; Kim et al., 2015; Hamed et al., 2020).

**TABLE 5:** Fibrinolytic enzymes produced by plants.

Plant	Name of enzyme	References
*Gliricidia sepium* PBSGS	Cysteine protease	da Silva et al. (2020)
*Ficus carica*	Serine protease	Hamed et al. (2020)
*Petasites japonicus*	Serine protease	Kim et al. (2015)
*Cnidoscolus urens* (L.)	Acid protease	De Menezes et al. (2014)
*Aster yomena*	Kitamase: Protease	Choi et al. (2014)
*Artocarpus heterophyllus*	Serine protease	Siritapetawee et al. (2012)
*Euphorbia hirta*	Hirtin: serine protease	Patel et al. (2012)
Chive (*Allium tuberosum*)	*Allium tuberosum* fibrinolytic enzyme (ATFE): Serine protease	Chung et al. (2010b)
Chive (*Allium tuberosum*)	ATFE-II: Serine protease	Chung et al. (2010b)

Other sources of fibrinolytic enzymes include snake venoms, earthworms, sponge, and parasites (Cintra et al., 2012; Chou et al., 2013; Fu et al., 2013; Girón et al., 2013; Fu et al., 2016; Verma and Pulicherla, 2017; Dhamodharan et al., 2019; Xavier et al., 2019). Snake venoms contain metalloproteases, which are fibrinolytic enzymes consists of a group of multigene protein families involved in many activities of fibrinolysis, hemorrhage, apoptosis, anti-coagulant anti-platelet and pro-coagulant effects (Sanchez et al., 2017). Metalloproteases could be obtained from the venoms of *Bothrops colombiensis*, *Trimeresurus mucrosquamatus*, and *Bathroxobin atrox* (Table 6). Snake venom metalloproteinases (SVMPs) are classified into four groups (PI to PIV) based on their domain structures. PI group snake venom metalloproteinases have molecular masses ranging from 20 to 30 kDa, contain only proteinase domain and have a weak hemorrhagic activity (Fox and Serrano, 2009). PII group contains both proteinase and disintegrin-like domains containing 30–60 kDa proteins. PIII group proteins comprised of a cysteine-rich domain, while PIV proteins include an additional lectin-like domain (Fox and Serrano, 2005). Snake venom metalloproteinases (SVMPs) are involved in fibrinolysis, in activating coagulation process through proteolytic activity, and in activating coagulation factors such as factor X and II. Furthermore, they are involved in inhibition or induction of platelet aggregation (Cintra et al., 2012). Besides serine proteases, earthworms such as *Lumbricus rubellus* and *Eisenia fetida* could produce lumbrokinase, a fibrin specific protease which could lower blood viscosity, minimizing platelet accumulation and promote thrombus dissolution by transporting them into the blood via intestinal epithelium (Fu et al., 2013; Verma and Pulicherla, 2017). Lumbrokinase exists as a complex of six serine protease isoforms, each isoform has different molecular weights ranging from 14 to 33 kDa with variety in fibrinolytic activity (Verma and Pulicherla, 2017). It has dual mechanism in clot degradation: acts on fibrin directly and activates plasminogen and convert it into plasmin, which subsequently induce plasmin-based clot dissolution (Wang et al., 2004). Another example of fibrinolytic enzyme from non-microbial organism is *Rhipicephalus (Boophilus) microplus* Cathepsin-L like 1 (BmCL1), which is isolated from *Rhipicephalus microplus*, a parasite grows on cattle and other animals. BmCL1 comprised of two isoforms with molecular weights of 26 and 22 kDa. It could degrade fibrinogen by hydrolyzing Aα- and Bβ-chains (Xavier et al., 2019).

**TABLE 6:** Fibrinolytic enzymes obtained from non-microbial sources.

Organism	Name of enzyme	References
Parasite (*Rhipicephalus microplus*)	BmCL1 (Rhipicephalus [Boophilus] microplus Cathepsin-L like 1); a protease	Xavier et al. (2019)
Sponge (*Agelas conifer*)	Protease from *Streptomyces radiopugnans* VITSD8	Dhamodharan et al. (2019)
Earthworm (*Pheretima posthumous*)	Serine protease	Verma and Pulicherla (2017)
Earthworm (*Lumbricus rubellus*)	Lumbrokinase	Fu et al. (2016)
Earthworm *Eisenia fetida*	Lumbrokinases (protease)	Fu et al. (2013)
*Bothrops colombiensis* venom	Colombienases: metalloproteinase	Girón et al. (2013)
*Trimeresurus mucrosquamatus* venom	TM-1	Chou et al. (2013)
*Bathroxobin atrox *snake venom	Batroxase: metalloproteinase	Cintra et al. (2012)

## Fibrinolytic Enzymes as Drugs for Treating Thrombin

### Streptokinase

Among the plasminogen activators, streptokinase (streptokinase) is the first drug approved by FDA for thrombosis treatment since 1950s. Streptokinase is a bacterial protein obtained from β-hemolytic streptococci of Lancefied groups A, C and G (Roohvand, 2018). The group C strain of *Streptococcus* equisimilis H46A (ATCC 12449 and ATCC 9542, introduced for streptokinase production in 1945 and 1992, respectively) have been widely used for production of streptokinase (Vadla et al., 2019). At present, streptokinase is available commercially under the trade names of Indikinase, Kabikinase, Varidase and Streptase (Roohvand, 2018).

**FIGURE 1:**
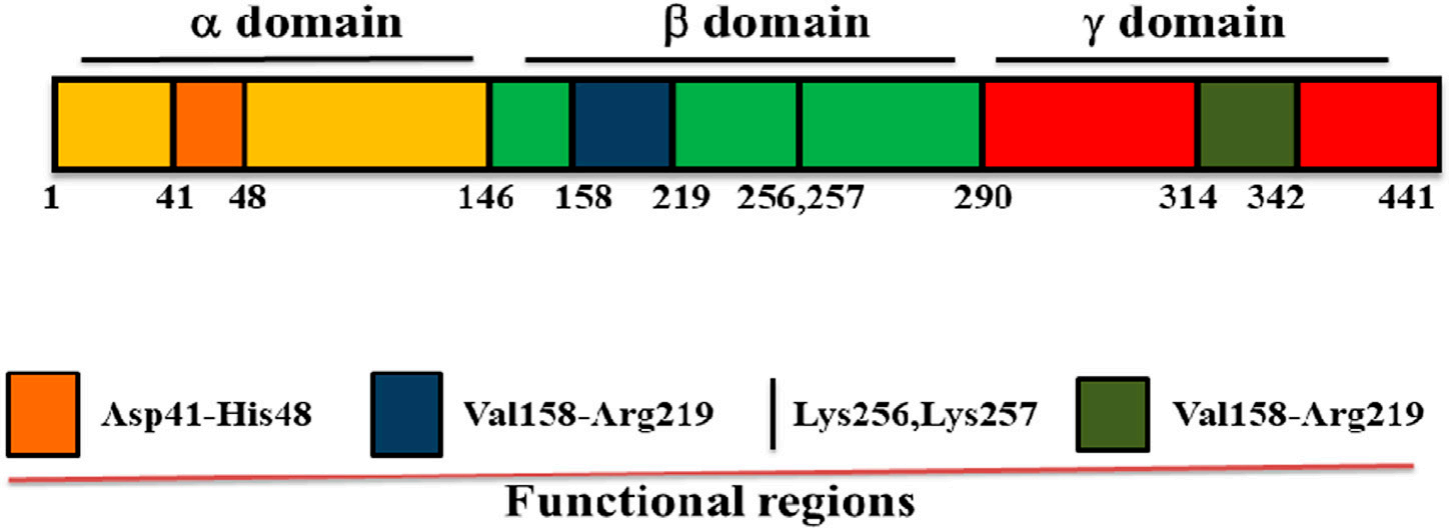
Domain structure of streptokinase. Streptokinase consists of three domains: α domain at 1–146, β domain at 147–290, and γ domain at 291–414 of the amino acid positions. It also contains many functional regions across the domain such as Asp41–His48 region between the first to 59th 59 amino acid residues of α domain, Lys256, Lys257, and Val158–Arg219 region of β domain, as well as Leu314–Ala342 region of γ domain.

Streptokinase is a single polypeptide enzyme made up of 414 amino acid residues with molecular weight of 47 kDa and performs its maximum activity at pH 7.5. It converts inactive plasminogen to plasmin through a series of protein-protein interactions that comes to an end with the formation of streptokinase-plasmin complex (Kazemi et al., 2019). Streptokinase does not contain cysteine, lipids, phosphorous and conjugated carbohydrates, and is made up from three structural domains: α domain at 1–146, β domain at 147–290 and γ domain at 291–414 amino acid positions linked by two flexible coil regions (Figure 1). There are many exosites or functional regions across the domains of streptokinase, including the Asp41–His48 region between 1-59 amino acid residues of α domain which controls its binding to plasminogen and activation (Sazonova et al., 2004; Verhamme and Bock, 2014), a 250 loop along with the pair of Lys 256, Lys 257 and Val158-Arg219 region of β domain which is responsible for plasminogen recognition, processing and streptokinase- plasmin complex formation (Tharp et al., 2009), and a coiled region of streptokinase γ domain (Leu314-Ala342) which regulates streptokinase-micro plasmin complex stabilization for plasminogen activation. Along with functional groups and exosites, streptokinase also contains hot spots or single residues for substitutions, such as V19F, V35E and S44K in its α domain, which play important role for the activation of streptokinase-plasmin complex (Aneja et al., 2013; Kazemi, et al., 2019).

Being a microbial protein, streptokinase is immunogenic and could trigger immune response. This limits its therapeutic potential, as it cannot be re-administered after its first use. Furthermore, its half-life in the blood flow is short. To overcome these limitations, studies have been made by using various strategies, including the structural modifications, chemical modifications, liposomal entrapment or encapsulation, and domain fusion (Roohvand, 2018). Structural modifications have been done using deletions, or substitutions of the amino acids. For example, two truncated streptokinase proteins (SK60-386 and SK143-386) showed enhanced fibrin-specific activity and lower immunogenicity compared to the full length streptokinase (Arabi et al., 2011); while substitution of Lys59 and/or Lys386 for glutamine increased its half-life (Adivitiya et al., 2018). Chemical modifications have been done by site specific or homogenous PEGylation or acylation (anisoylated plasminogen-streptokinase activator complex; APSAC) (Kunadian and Gibson, 2012). For instance, cysteine-specific thiol-mediated PEGylation of streptokinase increased its stability and half-life (Sawhney et al., 2016); while acylation of a complex consisting of human plasminogen and bacterial streptokinase (anisoylated plasminogen-streptokinase activator complex; APSAC) enhanced its specificity for blood clot (Ali et al., 2014). Modification in the delivery system of streptokinase, for example using liposomal entrapment or encapsulation of streptokinase in PEG or chitosan nanoparticles as well as platelet directed liposomes have also been explored (Vaidya et al., 2011; Baharifar et al., 2020; Hasanpour et al., 2021). These modifications not only could enhance its stability and half-life, but also could reduce its immunogenicity and improves its clot penetration properties. Another effort for improving streptokinase is domain fusion to produce chimeric and conjugated streptokinase proteins. Maheshwari *et al.* found that fusion of streptokinase with epidermal growth factor 4, 5, and 6 domains of human thrombomodulin reduced the risk of re-occlusion and hemorrhage (Maheshwari et al., 2016). Together, these modifications improve both the activity and drug ability of streptokinase, while reducing its side-effects.

### Staphylokinase

**FIGURE 2:**
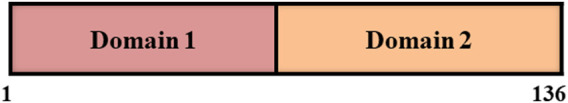
Domain structure of staphylokinase consisting two domains of equal size.

Staphylokinase is a bacterial protein that exerts its anti-thrombin activity by converting inactive plasminogen into active plasmin. It is found in the culture medium of many strains of *Staphylococcus aureus* (Gerheim, 1948; Lack, 1948). Initially, Davidson and Glanville precipitated protein containing staphylokinase by adjusting the pH to 3.3 with ∼10 mM HCl from supernatant fluid of cultures (Davidson, 1960; Glanville, 1963). SAK is a single polypeptide chain of 136 amino acids with molecular weight of 15.5 KDa, which has two domains of equal size with flexible dumbbell shape (Figure 2) (Bokarewa et al., 2006; Nedaeinia et al., 2020).

Staphylokinase does not directly degrade the fibrin. Indeed, it performs its activity in two- step mechanism. Firstly, a complex is formed between staphylokinase and plasminogen, then the active site of this complex is accessible to transform plasminogen into plasmin. After the formation of this complex, a peptide bond between lysine 10 and 11 of staphylokinase is hydrolyzed, and this in turn triggers the lysis of peptide bond between arginine 561 and valine 562 of plasminogen (Pulicherla et al., 2013). This results in the initial conversion of plasminogen to plasmin in staphylokinase-plasminogen complex. After small amount of plasmin has been synthesized, staphylokinase binds to plasmin, and finally this complex promptly and directly converts plasminogen to plasmin. The formation of these complexes, either to plasmin or plasminogen, is hampered by α2-antiplasmin in the absence of fibrin (Lijnen et al., 1992; Ram et al., 2013; Nedaeinia, et al., 2020). Thus, staphylokinase is fibrin specific and give good response to clot degradation and can be used potentially for the removal and treatment of blood clots (Nedaeinia, et al., 2020); however, its short half-life rendered its clinical use, and efforts are necessary to overcome this problem (Akhtar et al., 2017).

### Nattokinase

**FIGURE 3:**
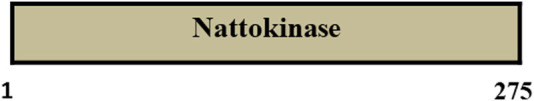
Domain structure of nattokinase consisting of a single polypeptide chain.

Nattokinase is a serine protease produced from the traditional Japanese food natto through the process of fermentation of soybeans with the bacterium *Bacillus subtilis*, and extracted for the first time by Sumi *et al.* in 1980s (Sumi et al., 1987; Nagata et al., 2017). It has a single polypeptide chain comprises of 275 amino acids with molecular mass of 27.7 KDa (Figure 3), and can work at the pH of 6–12 and temperature up to 60°C (Maeda et al., 2001).

Nattokinase increases the natural ability of the body to degrade the blood clots in different ways and confer many advantages such as oral administration, efficacy, cost effective, long lasting effects, stability in the gastrointestinal tract, as well as its potential to increase the ability of body to produce plasmin and urokinase (Kotb, 2012). It works by directly degrading the fibrin and plasmin substrate, transforms pro-urokinase into uPA, hydrolyses PAI-1 and enhance the level of tPA, which facilitate fibrinolytic activity (Sumi, et al., 1987; Yatagai et al., 2007; Chen et al., 2018). Furthermore, both *in vivo* and *in vitro* toxicology experiments have provided strong evidence about the safety of nattokinase for human oral consumption, as until now, there is no toxicity detected upon its usage (Lampe and English, 2016). Thus, nattokinase is a potential enzyme for dissolving blood clot, and has been extensively studied in Korea, Japan and China (Weng et al., 2017). Furthermore, its clinical trials for atherothrombotic prevention are being held in United States (Weng et al., 2017).

### Urokinase-Type Plasminogen Activator

**FIGURE 4:**
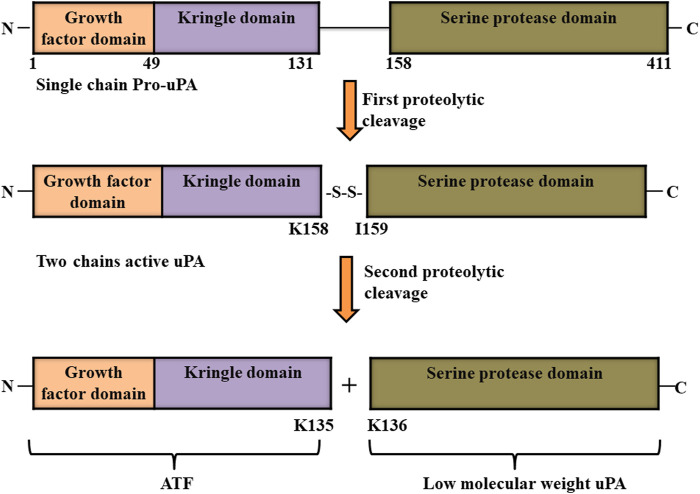
Structure of urokinase-type plasminogen activator (uPA). Pro-uPA is secreted as an inactive single polypeptide chain consisting of a growth factor domain, kringle domain and serine protease domain, and undergoes first proteolytic cleavage between its Lys158 and IIe159. A second-round cleavage at the peptide bond between its Lys135 and Lys 136 totally cleaves the two chains of uPA into two parts: the inactive amino-terminal fragment (ATF) and active low molecular weight form of uPA (Mahmood et al., 2018).

Urokinase plasminogen activator, which is also known as urokinase, was first identified in 1947 in urine as unnamed novel fibrinolytic protein by MacFarlane and Pilling (1947) before being named as urokinase half a decade later (MacFarlane and Pilling, 1947; Sobel et al., 1952). It is produced and secreted as an inactive single polypeptide chain of 411 amino acids called pro-urokinase or pro-uPA from macrophages, endothelial cells, some tumor cells, and renal epithelial cells. Urokinase is comprised of three domains: growth factor domain (GFD, from 1 to 49 amino acids in the pro-uPA protein) and kringle domain (KD, from 50 to 131 amino acids) which are both located at the N- terminus, as well as a serine protease domain (P, from 159 to 411 amino acids) at the C-terminus of the pro-uPA protein. The N-terminal and C-terminal regions of pro-uPA are linked by a linker region (from 132 to 158 amino acids). After being secreted, pro-uPA goes through a two-rounded proteolytic process that cleaves it between Lys158 and IIe159 at the linker region; however, the two chains formed are still linked through disulfide bond. Two chains uPA has a molecular weight of 54 kDa. A second round cleavage at the peptide bond between Lys135 and Lys 136 totally cleaves the two chain of uPA into two parts: the inactive amino-terminal fragment (ATF) that contains growth factor domain and kringle domain, and a low molecular weight (33 kDa) active form of uPA with serine protease domain (Figure 4) (Poliakov et al., 2001). Finally, due the presence of GFD and amino-terminal fragment (ATF), pro-uPA as well as the two chain forms of uPA (active serine protease domain and inactive ATF with growth factor domain and kringle domain) binds to its receptor (uPAR) with similar affinity. This binding in turn enhances the conversion of plasminogen into plasmin (Blasi and Carmeliet, 2002; Mahmood et al., 2018; Kadir and Bayraktutan, 2020). Owing to its high activity as a plasminogen activator, urokinase is a potential drug approved by FDA for clinical use for the treatment of cardiovascular diseases (Akhtar et al., 2017).

### Recombinant Tissue-Type Plasminogen Activator

**FIGURE 5:**
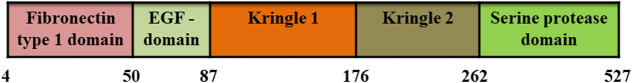
Domain structure of recombinant tissue-type plasminogen activator (rtPA). It consists of fibronectin type I domain, an epidermal growth factor domain, as well as kringle 1, kringle 2 and serine protease domains.

Tissue plasminogen activator is a serine protease with fibrin specific thrombolytic activity. It has a molecular weight of 70 kDa and consisting of 527 amino acids. It comprises of five domains: i) a fibronectin type I domain at N-terminal with 47 amino acid residues (F, 4–50 residues), ii) an epidermal growth factor domain (EGF, residue 50–87), iii) kringle 1 (K1, 87 to 176 residues), iv) kringle 2 (K2, 176–262 residues), and v) a serine protease domain (P, residues 276–527) (Figure 5) (Lee and Im, 2010; Nedaeinia, et al., 2020). The activity of plasminogen activator is weak in the absence of fibrin; while the presence of fibrin significantly increased its activity. Furthermore, its activity is inhibited *in vivo* by PAI-1 and the amino acids at 296–299 are crucial for this inhibition (Ram et al., 2013). To overcome such drawbacks, different forms of recombinant tPA, including Alteplase, Reteplase, Tenecteplase, and Desmoteplase have been developed using recombinant DNA technology. Among them, Tenecteplase and Reteplase are approved for clinical use (Wander and Chhabra, 2013); however*,* they still have side effects of bleeding complications, fibrin specificity and allergic reactions (Ram et al., 2013). Therefore, currently, many researchers are focusing on reducing these side effects and increasing efficacy of these fibrinolytic enzymes. For example, magnetic nanoparticles-based dual targeted delivery strategy (peptide/rtPA conjugated poly(lactic-co-glycolic acid) (PLGA) magnetic nanoparticles (PMNPs)) increased the fibrin specificity of rtPA (Ram et al., 2013; Chen et al., 2020; Nedaeinia, et al., 2020); while Güner et al. used the strategy of prolonged thrombolytic therapy with low-dose and slow-infusion of tissue-type plasminogen activator. This strategy reduced the bleeding complications to a significant level (Güner et al., 2020). Taken together, if the above mentioned side effects are controlled, rtPAs could be promising drugs for thrombolytic therapy.

## Discussion

**FIGURE 6:**
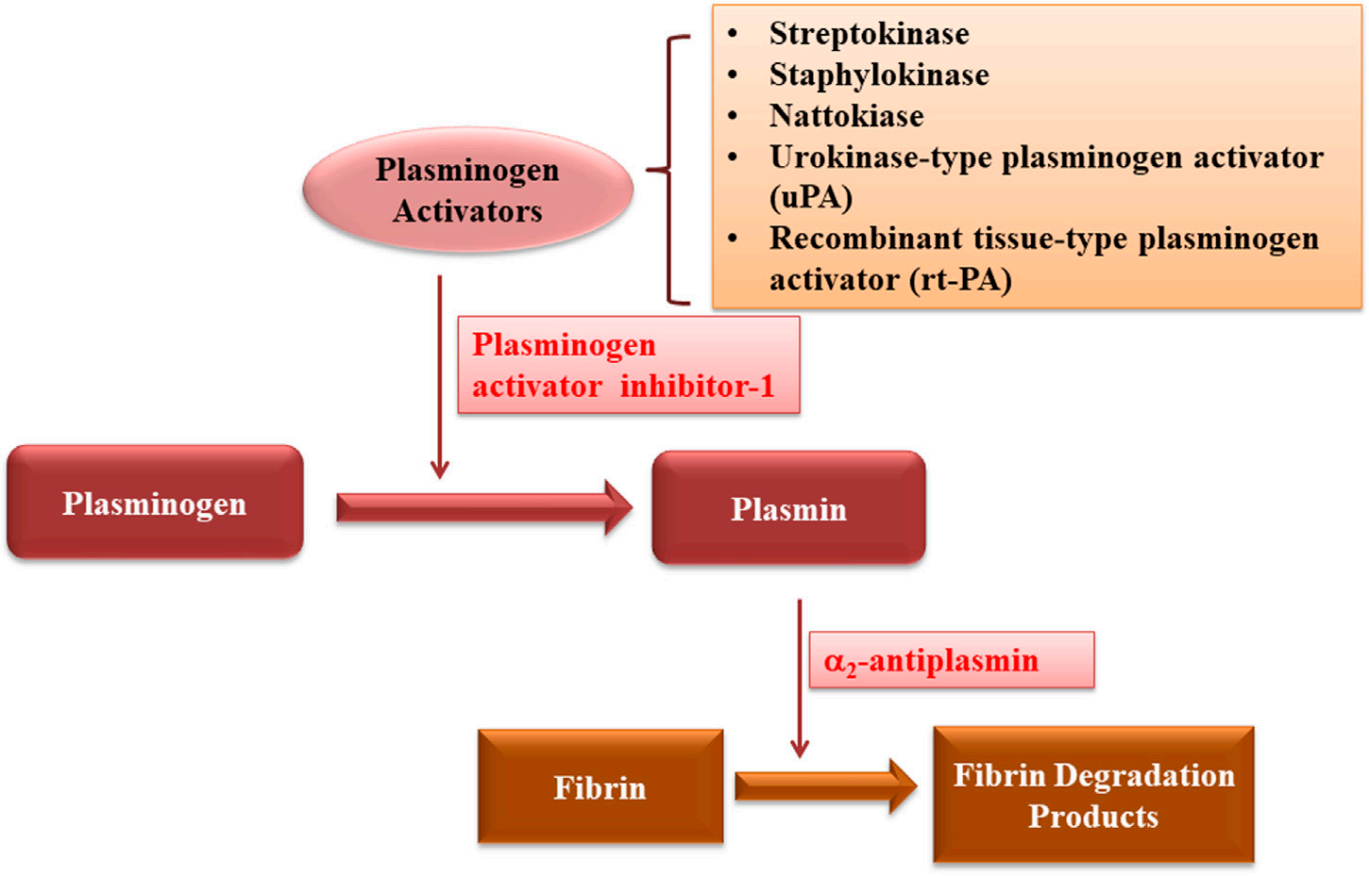
Schematic diagram showing the mechanism of action of thrombolytic drugs.

Since its discovery, various fibrinolytic enzymes have been discovered from different sources, and have been used for the thrombolytic therapy (Figure 6; Table 7). They are reducing the risk of morbidity and mortality rates related to stroke, myocardial infarction and cardiovascular diseases. Various kinds of fibrinolytic enzymes, such as Streptokinase, Urokinase, Recombinant tissue plasminogen activator (rtPA), Reteplase, and Tenecteplase have been used for clinical application and are commercially available. While they exhibit benefits due to their fibrin specificity, prolonged plasma half-life, stability, resistance to PAI-1, less antigenicity, cost effectiveness and low bleeding complications, efforts are still needed to enhance their half-life, fibrin specificity, efficacy, biocompatibility and resistance to inhibition by plasma inhibitors as well as to reduce major side effects of bleeding and hemorrhage.

**TABLE 7:** Comparison of different thrombolytic drugs.

Thrombolytic drugs	Molecular weight (kDa)	Number of amino acids	Domain structure	Fibrin specificity	Plasminogen activation
Streptokinase	47	414	3 (α, β, and γ)	No	Indirect
Staphylokinase	15.5	136	2 domains	Yes	Indirect
Nattokinase	27.7	275	1 domain	Yes	Direct
Urokinase-type plasminogen activator	54-33	411	3 (GFD, KD, P)	No	Direct
Recombinant tissue-type plasminogen activator	70	527	5 (F, EGF, K1, K2, P)	Yes	Direct

Among different sources, microbial fibrinolytic and thrombolytic enzymes have gathered more medical attention in previous years for their low-cost and large-scale production. Some of the microbial fibrinolytic such as Streptokinase, Urokinase, Recombinant tissue plasminogen activator (rtPA), Reteplase, and Tenecteplase have been approved for their clinical use. These drugs are potential to reduce the risk of death or reccurence of blood clots; however, their side effects, including major and minor haemorrhagic events, hemorrhagic transformation, brain edema, and stroke, have also been reported. There are also many other fibrinolytic enzymes from various sources that have been proven to be effective for thrombolytic therapy *in vitro*, and thus can also be used as potential drugs for anti-thrombotic therapy; however, their *in vivo* experimental studies and immunogenicity-based analysis are yet to be done.

In summary, utilizing fibrinolytic enzymes for anti-thrombolytic therapy is promising. However, while different approaches have been used to reduce their side effects, efforts are still needed to overcome the problems associated with bleeding, hemorrhage, and allergic reactions. Furthermore, further optimization of their production process is also needed in order to design safe and cost-effective drugs. Nevertheless, the use of fibrinolytic enzymes for thrombolytic therapy will be more promising and far better option in future.
